# Dual Cardiac Arrests Leading to Hypercoagulability and Extensive Upper Extremity Deep Vein Thrombosis: A Hematological Case Report

**DOI:** 10.7759/cureus.61100

**Published:** 2024-05-26

**Authors:** Eli Zolotov, Neria Bitton, Anat Sigal, David Kim, Caden Quintanilla

**Affiliations:** 1 Internal Medicine, Hackensack University Medical Center, Hackensack, USA; 2 Internal Medicine, Geisinger Medical Center, Danville, USA; 3 Pediatrics, Hackensack University Medical Center, Hackensack, USA

**Keywords:** hematology, hypercoagulability, blood hypercoagulability, upper extremity dvt, deep vein thrombosis (dvt), acute dvt, out-of-hospital cardiac arrest

## Abstract

Upper extremity (UE) deep vein thrombosis (DVT) is a rare yet significant complication that can occur following cardiac arrest (CA). CA initiates a prothrombotic state via various processes, including stasis, endothelial damage, and an impaired balance between thrombogenesis and fibrinolysis, which may contribute to UE DVT formation. Inadequate cardiopulmonary resuscitation (CPR) in the field may further exacerbate blood stasis and clot formation.

This case report describes an 80-year-old male with a history of bladder cancer who experienced two cardiac arrest events and subsequently developed an extensive left UE DVT. Despite treatment with a heparin drip and other supportive measures, the patient’s condition deteriorated, and he passed away on the tenth day of hospitalization. This case is the first to describe UE DVT post-CA. It underscores the importance of recognizing and proactively managing hypercoagulable states post-CA, which can lead to significant DVTs in atypical locations that may evolve into life-threatening conditions.

## Introduction

Upper extremity (UE) deep vein thrombosis (DVT) is a condition characterized by thrombus formation in the deep veins, affecting the blood drainage of the upper extremities. It accounts for approximately 1-4% of all DVT cases [1]. The condition is categorized into primary and secondary types. Primary UE DVT occurs spontaneously, while secondary UE DVT is associated with specific precipitating factors, such as prothrombotic states and catheter-related complications. The development of DVT can be understood through the framework of Virchow's triad: venous stasis, hypercoagulability, and endothelial dysfunction [2].

Cardiac arrest (CA), particularly in an out-of-hospital setting, predisposes patients to DVT due to its impact on each component of Virchow's triad. First, inadequate cardiopulmonary resuscitation (CPR) may result in blood stasis, leading to poor circulation and an increased risk of thrombus formation in various body regions, including the heart chambers, lungs, and extremities [3,4]. Second, patients who experience CA often exhibit significant thrombogenesis and impaired fibrinolysis [5]. Lastly, post-CA, increased catecholamine release can contribute to endothelial dysfunction, further exacerbating the prothrombotic state [6].

This case report presents an 80-year-old male with a past medical history of hypertension and bladder cancer who experienced two cardiac arrests and subsequently developed a left UE DVT with suspected metastatic disease. This case is the first to underscore the association between CA and UE DVT formation.

## Case presentation

An 80-year-old male with a past medical history of hypertension, depression, and bladder cancer (status post-resection chemotherapy three years ago in Venezuela) was found unresponsive at home. Emergency Medical Services (EMS) noted severe hypoglycemia (<20 mg/dL), hypotension, a locked jaw, and foaming at the mouth. The patient experienced a CA shortly afterward, requiring CPR. Return of Spontaneous Circulation (ROSC) was achieved after eight minutes, and he was intubated in the field.

Upon arrival at the emergency room (ER), the patient was sedated, intubated, and ventilated. His initial vital signs included a blood pressure of 151/132 mmHg, a heart rate of 87 beats per minute, a temperature of 95.8°F, and 100% oxygen saturation while being manually bagged. Thirty minutes later, the patient experienced a second CA, and after 10 minutes of CPR, ROSC was achieved, and he was admitted to the ICU.

**Figure 1:**
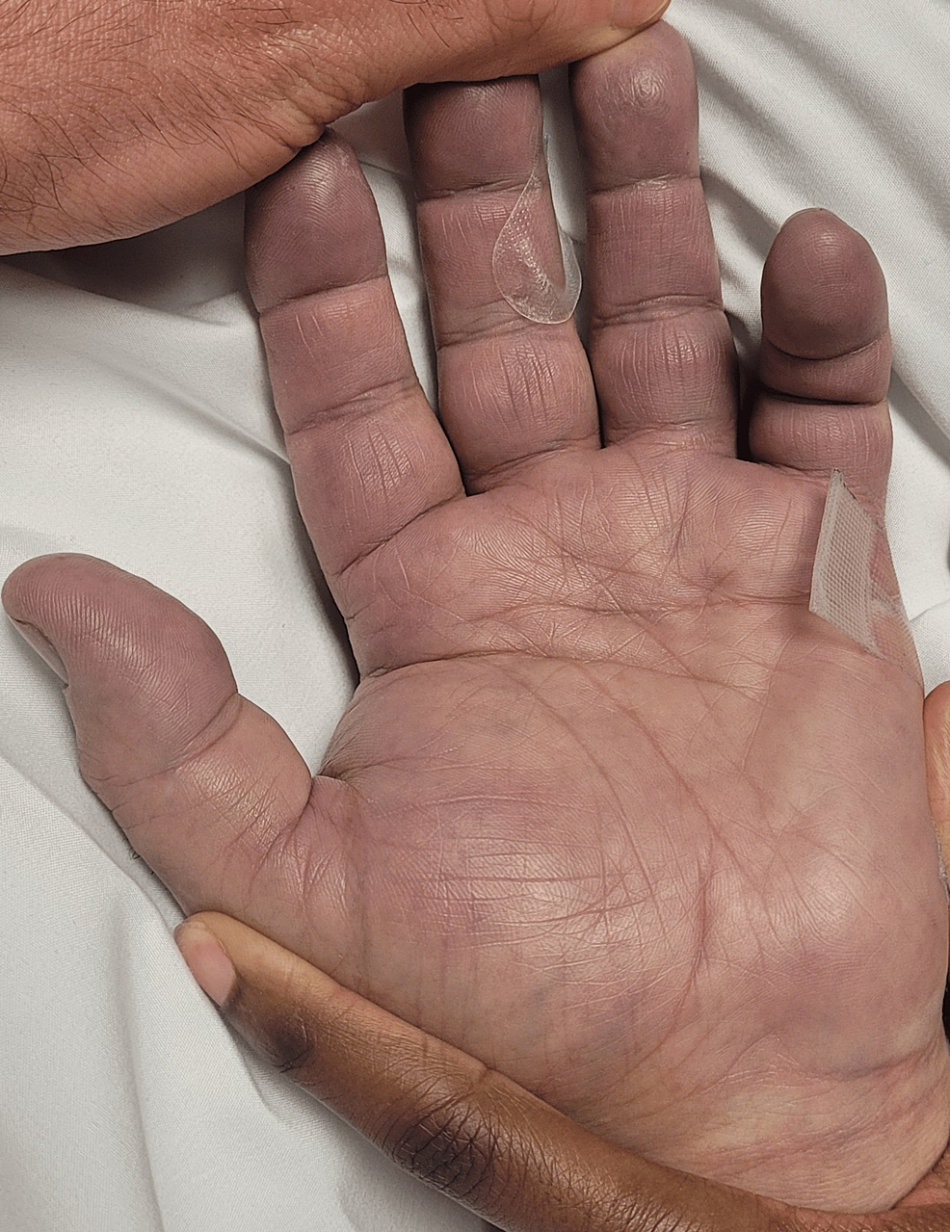
Tense and edematous left hand extending to the wrist, with ecchymosis of the distal digits.

**Figure 2:**
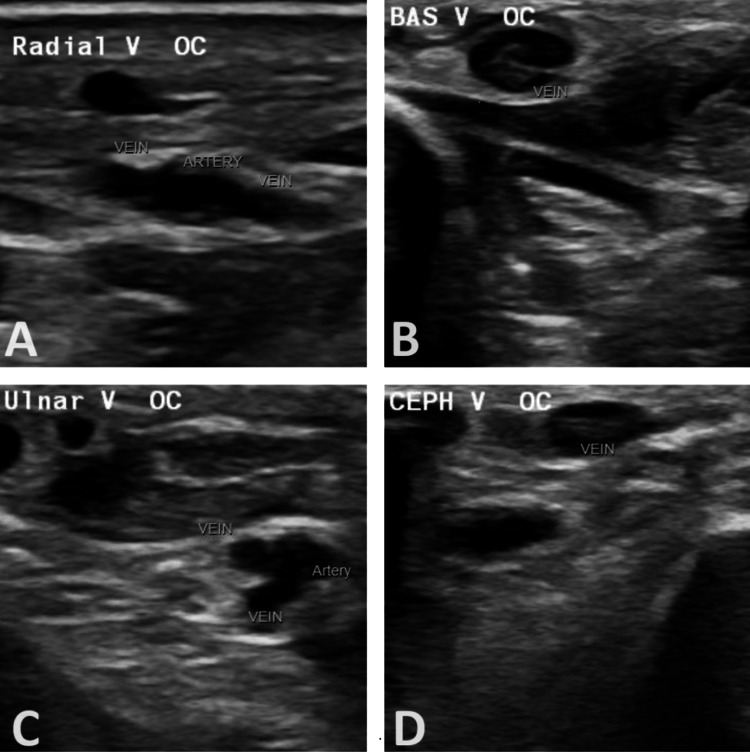
Venous doppler of the upper extremity. Occlusion of the distal upper arm and forearm radial [A], forearm basilic [B], forearm ulnar [C], mid-to-distal upper arm and forearm cephalic veins [D] by echolucent thrombi.

His labs taken during these events and six hours later are summarized in Table 1. Physical examination revealed an intubated, sedated patient with dilated pupils, equal and reactive to light, and coarse breath sounds. His left hand was swollen, ecchymotic, cool to the touch, and tense, with a palpable radial pulse (Figure 1). Duplex ultrasonography showed a patent arterial supply but identified radial venous thrombosis. Doppler imaging revealed deep vein thrombosis (DVT) involving the left radial, ulnar, basilic, and cephalic veins (Figure 2). The venous Doppler and arterial duplex of the right UE and bilateral lower extremities were negative for further vascular compromise, or DVT. After consulting with the vascular team and hand surgeons, acute surgical intervention was not recommended. Instead, the patient was placed on a heparin drip for the acute upper extremity DVT.

**Table 1:** Laboratory data on admission and six hours later.

Variable	Reference Range	Admission	6 Hours Later
Hematology			
Hemoglobin (g/dL)	13.0 - 17.0	9.5	8.9
Hematocrit (%)	36 - 46	36.8	30.7
White-cell count (per μL)	4,000 - 11,000	23,900	14,900
Neutrophils (%)	40 - 75	14.3	13.4
Lymphocytes (%)	13 - 43	17	4
Monocytes (%)	0 - 13	4	1
Eosinophils (%)	0 - 5	1	0
Platelet count (per μL)	135,000 - 430,000	406,000	385,000
Chemistry			
Sodium (mmol/L)	136 - 145	145	141
Potassium (mmol/L)	3.5 - 5.1	6.7	4.4
Carbon Dioxide (mmol/L)	22 - 29	15	14
Creatinine (mg/dL)	0.3 - 1.5	1.88	1.54
Aspartate aminotransferase (U/L)	5 - 34	2,259	>4,202
Alanine aminotransferase (U/L)	0 - 55	1,240	2,099
Total bilirubin (mg/dL)	0.2 - 1.2	0.7	0.7
Alkaline phosphatase (IU/L)	43 - 122	176	152
Glucose (mg/dL)	82 - 115	70	220
Lactate (mmol/L)	0.5 - 2.0	22.8	14.7
Arterial Blood Gas			
pH	7.350 - 7.450	7.010	7.416
pCO2 (mmHg)	35.0 - 45.0	44.9	40.8
pO2 (mmHg)	75.0 - 100.0	214.1	254.3

Additional imaging included a chest X-ray, which showed a right pulmonary infiltration suggestive of pneumonia. Urinalysis revealed an infection with *Escherichia coli*, and the patient was treated with piperacillin-tazobactam (4.5 g three times daily for five days) and ceftriaxone (for two additional days). The patient’s non-purposeful movements prompted a neurological evaluation with a CT of the head and a vEEG, which showed no acute pathology. However, an MRI revealed punctate acute infarctions in the left centrum semiovale and right parietal lobe, along with a small area of leptomeningeal enhancement in the left cerebellar folia. Given that the patient was outside the window for receiving tPA, he was started on aspirin (81 mg daily) and atorvastatin (80 mg daily).

A CT angiogram of the chest, abdomen, and pelvis ruled out pulmonary embolism or aortic dissection but highlighted extensive metastatic disease, including multiple small bilateral pulmonary metastases, a right renal infiltrative neoplasm with satellite metastases extending to the posterior right hepatic lobe, retroperitoneal lymphadenopathy, and multiple lytic osseous metastases, most pronounced in the pelvis. The oncology team was consulted, but the patient declined further diagnostic studies and antineoplastic therapy, leaving the type of malignancy unclear.

Despite initial improvement leading to extubation on the seventh day of hospitalization and partial resolution and recanalization of the left UE DVT on Doppler performed on the 10th day, the patient’s respiratory and neurological status deteriorated on the 10th day, and he passed away.

## Discussion

This case report is the first to clearly describe UE DVT formation following CA. It emphasizes the importance of recognizing the hypercoagulable state post-CA, which warrants attention comparable to that given to lower extremity DVT [7].

In cases of out-of-hospital CA, the quality of CPR is often compromised. Inadequate CPR can exacerbate blood stasis and increase the risk of clot formation. In this case, the patient underwent two rounds of CPR, likely worsening stasis and contributing to the development of the UE DVT [3,4]. Moreover, CA can lead to an imbalance between thrombogenesis and fibrinolysis [5]. CA has been shown to trigger the coagulation cascade while impairing the body's ability to break down clots. A prospective study by Fatovich et al. suggested that an early bolus of tenecteplase may increase the ROSC in CA patients. However, further evaluation showed that tenecteplase use did not significantly improve ROSC rates, survival, or neurological outcomes. Therefore, its use should be tailored to individual patient circumstances [8-10]. While some consider hypothermia a potential contributor to a hypercoagulable state, data remains inconsistent regarding its impact on thrombogenesis [11,12].

Another reported case documented a patient with a history of coronary artery disease who developed UE DVT and subsequently experienced a fatal CA [13]. Unlike our case, where the DVT was identified post-CA, this patient developed DVT before the CA. Furthermore, postmortem autopsy findings suggested that the patient's CA could have been secondary to a pulmonary embolism. Although the sequence of events differs from ours, it highlights the critical role of UE DVT as a potential precursor to pulmonary emboli, which can precipitate CA and complicate resuscitation efforts [14].

In our case, the suspected underlying malignancy cannot be disregarded and may contribute to the patient's hypercoagulable state. The patient’s imaging, including CT and MRI, revealed multiple masses in the body, including demonstrated leptomeningeal enhancement, indicating potential CNS metastasis. While further diagnostic evaluations, including biopsies, were not performed, extensive metastatic disease remains high on the differential. Cancer-related hypercoagulability is a well-documented concern in cancer patients due to fibrinolytic and procoagulant activity. The production of cytokines and the physical interaction of tumor cells with different blood cells and the endothelium may further contribute to the hypercoagulable state [15].

## Conclusions

This case is the first to describe the formation of a new UE DVT post-CA. It raises awareness of hypercoagulable states post-CA, which may lead to the formation of significant DVTs in atypical locations such as the upper extremities. These complications must be seriously considered, as they can lead to life-threatening conditions such as pulmonary embolism and cardiac arrest. This case underscores the complex interplay of factors contributing to thrombogenesis post-cardiac arrest and highlights the critical need for monitoring and proactive management of DVT in this vulnerable population.
